# Individualized anemia management enhanced by ferric pyrophosphate citrate protocol

**DOI:** 10.1038/s41598-022-23262-1

**Published:** 2022-11-22

**Authors:** Yossi Chait, Brian H. Nathanson, Michael J. Germain

**Affiliations:** 1 Mechanical and Industrial Engineering Department, University of Massachusetts, Amherst, MA 01003 USA; 2 OptiStatim, LLC, Longmeadow, MA 01106 USA; 3 Renal and Transplant Associates of New England, PC, Springfield, MA 01107 USA; 4 Division of Nephrology, Baystate Medical Center, Springfield, MA 01199 USA; 5 University of Massachusetts Medical School, Worcester, MA 01655 USA

**Keywords:** Medical research, Nephrology

## Abstract

The optimal use of erythropoiesis-stimulating agents (ESAs) and parenteral iron in managing anemia in end-stage renal disease (ESRD) remains controversial. One-size-fits-all rule-based algorithms dominate dosing protocols for ESA and parenteral iron. However, the Food & Drug Administration (FDA) guidelines for using ESAs in chronic kidney disease recommend individualized therapy for the patient. This prospective quality assurance project was at a single hemodialysis (HD) center comprising three 6-month phases (A, B, C) separated by 3-month washout periods. Standard bi-weekly ESA dose titration and intravenous (IV) iron sucrose protocols were used in baseline Phase A, and ferric pyrophosphate citrate (FPC) augmented iron in Phase B. In Phase C, an FPC protocol and weekly, individualized ESA management were used. We examined clinic-level mean differences in hemoglobin (Hb) and ESRD-related outcomes by phase with repeated ANOVA. To examine the Hb at the patient level, we used multi-level mixed-effect regression adjusting for phase, month, and other relevant confounders at each month over time to derive the mean marginal effects of phase. There were 54, 78, and 66 patients in phases A, B, and C, respectively, with raw mean Hb values of 9.9, 10.2, and 10.3 g/dL. The percentage of Hb values < 9 g/dL declined from 14.3% in Phase A to 7.6% in Phase C (p = 0.007). The multivariable mixed-effect regression results showed mean marginal Hb was higher by 0.3 mg/dL and 0.4 mg/dL in Phases B and C, respectively, compared to Phase A. We also observed reduced ferritin (p = 0.003) and transferrin saturation (TSAT) (p = 0.008) levels from Phase A to Phase C with the repeated ANOVA analysis. Ferric pyrophosphate citrate (FPC) appears to support more efficient ESA-stimulated erythropoiesis. Moreover, individualized ESA management combined with FPC (Phase C) was associated with further improvement in efficiency as we observed the fewest patients with Hb values < 9 g/dL concurrent with greater decreases in ferritin levels and reduced ESA doses. However, future prospective studies to confirm these findings on a larger, more diverse cohort of ESRD patients are warranted.

## Introduction

Anemia is one of the most common complications of End-Stage Renal Disease (ESRD)^1^. Standard anemia care in ESRD involves treating patients with erythropoiesis-stimulating agents (ESAs) and parenteral iron^2^. Anemia management protocols (AMPs) guide the titration of ESA and iron in response to periodic hemoglobin (Hb) measurements and iron parameters. These guidelines are typically population-based, one-size-fits-all, "if–then" decision rules. However, the recent KDIGO conference on controversies in optimal anemia management concluded: “…optimal thresholds, targets, and treatment strategies for anemia remain unknown, and have not been customized… The need for increasing the complexity and specificity of treatment goals for patients is in keeping with trends to individualize therapy…”^3^.

Homeostasis in the human body maintains physiologic parameters within the desired ranges in the face of changing environments, namely, external stimuli, pathologic processes, and variations in internal behavior. Homeostasis relies on feedback loops to meet its goals^4^. For example, erythropoiesis is a homeostatic process in which the body maintains iron and erythropoietin levels to meet its needed oxygen delivery to tissues via feedback loops. Unfortunately, no relevant mathematical models describe the dynamics of Hb stability in response to ESA and intravenous (IV) iron therapy.

Effective anemia management in ESRD remains an open problem due to the heterogeneous dose–response profile within the population. Furthermore, the impact of variables that could affect Hb in ESRD can change over time (e.g., vitamins, gastrointestinal bleeding, transfusions^5^, fluid status, infection status, hospitalization status, and the precision and accuracy of laboratory assays)^6^. A critical but often overlooked aspect of all AMPs based either on predictive models or control design is their feedback interaction with erythropoiesis forming a closed-loop system, i.e., a homeostatic system. The performance of a closed-loop (homeostatic) system can only be inferred from feedback principles^7,8^.

ESA and parenteral iron dosing are decoupled, which is in stark contrast to the physiology of erythropoiesis. The administration of parenteral iron is essential to compensate for an annual iron loss of 2–3 g in hemodialysis (HD) patients^9^ and to match the non-uniform demand of ESA-stimulated erythropoiesis. Oral iron is thought to be inadequate in dialysis due to poor absorption and tolerance issues, although some small studies with different oral iron products have shown efficacy. The optimal dosing of parenteral iron is challenging. No studies have determined the optimal level of routinely measured iron parameters to distinguish functional from absolute iron deficiency. Current iron protocols focus on bringing ferritin and transferrin saturation (TSAT) within certain target ranges. Unfortunately, there is no consensus about ferritin and TSAT levels defining iron deficiency in HD patients^10^. In addition, iron repletion dosing is not synchronized with short-term iron demands in the bone marrow caused by transient ESA-stimulated erythropoiesis^3^.

The PIVOTAL trial^11^ showed that a proactive high-dose IV iron (iron sucrose) strategy could raise Hb levels and reduce ESA demand in iron-depleted ESRD patients. However, data from United States (US) centers participating in the Dialysis Outcomes and Practice Patterns Study (DOPPS) suggest that a liberal IV iron approach is already being realized in the US^12^. Nevertheless, whether a more aggressive approach is safe and effective remains uncertain^12,13^.

A novel approach utilizing a novel iron formulation, ferric pyrophosphate citrate (FPC, Triferic), delivered via dialysate during hemodialysis, was shown to effectively replace iron lost during HD without a significant increase in iron parameters. In randomized clinical trials, it also exhibited a similar safety profile to a placebo and maintained Hb levels^10,14^. These trials suggested that using FPC effectively replaces average HD-related iron losses on a per-treatment basis. Our prospective quality improvement project was designed to reduce unwanted variability with a five-phased process (define, measure, analyze, improve, control). We observed changes in anemia-related variables following the implementation of a FPC-protocol and an individualized ESA protocol (previously described in the literature^8^).

## Methods

This was a three-phase, prospective quality improvement project at a single USA hemodialysis (HD) center from July 2016 to June 2018. Each phase was six-month-long with a three-month washout period between phases. In the first baseline phase (Phase A), standard bi-weekly ESA (Aranesp™) dose titration and parenteral IV iron sucrose (Venofer) protocols (Supplementary material, Appendix A) were followed based on bi-weekly Hb measurements and monthly iron parameters measurements. In the second phase (Phase B), parenteral IV iron was replaced by a ferric pyrophosphate citrate protocol (FPC, Triferic™) (Supplementary material, Appendix A). Finally, in Phase C, the FPC-protocol was maintained, and ESA dose titration was handled using a weekly, individualized ESA management protocol (Supplementary material, Appendix A). Parenteral IV iron was administered during the FPC protocol when iron parameters were below target. The Hb target in phases A and B was 9–11 g/dL while minimizing < 9 and > 11 values per a standard anemia protocol, and 10.2 g/dL in Phase C per the individualized ESA protocol. All patients receiving maintenance hemodialysis at the clinic were included in the project. An exemption, including an informed consent waiver, was approved by an institutional review board (IRB) for this quality improvement project criteria (Quorum IRB Seattle, WA QR #: 32054). All methods were carried out in accordance with relevant guidelines and regulations for dialysis units.

Patient data were summarized by means and standard deviations for continuous variables and counts and percentages for categorical variables. To compare independent means, we used Student’s t-test, and to compare independent proportions, we used the Chi-square test. To examine results at the clinic level, we assessed the overall means of the variables of interest (Hb, TSAT, ferritin, etc.) at each month by the three Phases using a repeated ANOVA model with Phase and month order (i.e., 1 to 6) modeled as main effects and with an interaction term between them. p-values for the phase association on the outcome were adjusted with the Box correction factor, and p-values for the pair-wise combinations in the repeated ANOVA model were derived using the Tukey–Kramer method. Next, using multi-level mixed-effect regression (which considers the same patients measured over time), with Hb as the dependent variable, we included as predictors the variables: phase, month, the interaction between phase and month, ferritin, TSAT, total iron-binding capacity (TIBC), Aranesp, and Total parenteral IV iron at each month over time. From this model, the marginal effects of Phase were derived and plotted.

## Results

A total of 93 HD patients participated in the project though not all patients were present in each Phase: 54, 78, and 66 patients in Phases A, B, and C, respectively. These numbers represent the average turnover of patients in this clinic. No patient had adverse reactions to ferric pyrophosphate citrate. Patient characteristics are presented in Table 1. Briefly, the cohort had a mean (SD) age of 62.0 (15.3) years, and gender was approximately equally split: 28.0% were self-reported as Caucasian, 37.6% were Latino/Hispanic, and 22.6% were African American/Black.

**Table 1:** Patient characteristics.

Variable	Mean (SD) or n (%); N = 93 (all patients)	Phase A N = 54	Phase B N = 78	Phase C N = 66
Age	62.0 (15.3)	62.9 (14.0)	62.5 (14.7)	62.0 (16.4)
Male Gender	46 (49.5%)	28 (51.9%)	41 (52.6%)	32 (48.4%)
**Race**				
Caucasian/White	26 (28.0%)	17 (31.5%)	21 (26.9%)	17 (25.8%)
African American/Black	21 (22.6%)	12 (22.2%)	18 (23.1%)	14 (21.2%)
Latino/Hispanic	35 (37.6%)	21 (38.9%)	31 (39.7%)	25 (37.9%)
Asian	7 (7.5%)	3 (5.6%)	7 (9.0%)	6 (9.1%)
Other/Unknown	4 (4.3%)	1 (1.9%)	1 (1.3%)	4 (5.0%)
Diabetes (Type 1 or Type 2)	55 (59.1%)	37 (68.5%)	50 (64.1%)	37 (56.1%)

The means and SDs at the clinic level at each phase (i.e., mean of monthly means for each patient at a given phase) are shown in Table 2. The monthly transfusion rate, defined as the number of monthly events per patient census, was: 3.87%, 3.24%, and 0.47% in Phases A, B, and C, respectively. Mean hemoglobin was 9.9, 10.2, and 10.3 g/dL, mean Ferritin was 1005, 885, and 765 mg/L, mean transferrin saturation (TSAT) was 36.2, 28.5, and 29.8%, mean monthly Aranesp doses was 97, 95, and 90 mcg, and mean monthly total parenteral iron (iron sucrose and/or ferric pyrophosphate citrate) was 146, 145, and 150 mg.

**Table 2:** Repeated ANOVA analysis at the clinic level.

Variable	Phase A	Phase B	Phase C	p-value that the 3 phases are different	p-value that Phase A = Phase B	p-value that Phase A = Phase C	p-value that Phase B = Phase C value
Hb (g/dL)^1,2^	9.9 (0.2)	10.2 (0.3)	10.3 (0.1)	0.032	0.022	0.011	0.323
TSAT (%)^1,2^	36.2 (3.5)	28.5 (1.1)	29.8 (1.4)	0.009	0.004	0.008	0.279
Ferritin (mcg/L)^1,2,3^	1005 (45)	885 (57)	765 (83)	0.008	0.023	0.003	0.024
Serum iron (mcg/dL)^1,2^	82 (9)	65 (3)	69 (3)	0.016	0.007	0.012	0.278
TIBC (mcg/dL)^1,2^	225 (3)	230 (2)	232 (2)	0.021	0.017	0.008	0.216
Aranesp (mcg)	97 (17)	95 (14)	90 (12)	0.532	0.691	0.279	0.444
Venofer (mg)^1,2^	146 (56)	61 (49)	68 (30)	0.068	0.025	0.031	0.761
Triferic (mg)	NA	88 (4)	87 (5)	NA	NA	NA	NA
Venofer + Triferic (mg)	146 (56)	145 (48)	150 (29)	0.4891	0.966	0.225	0.804

^1^indicates a statistically significant difference between Phase A and Phase B.

^2^indicates a statistically significant difference between Phase A and Phase C.

^3^indicates a statistically significant difference between Phase B and Phase C.

**Figure 1:**
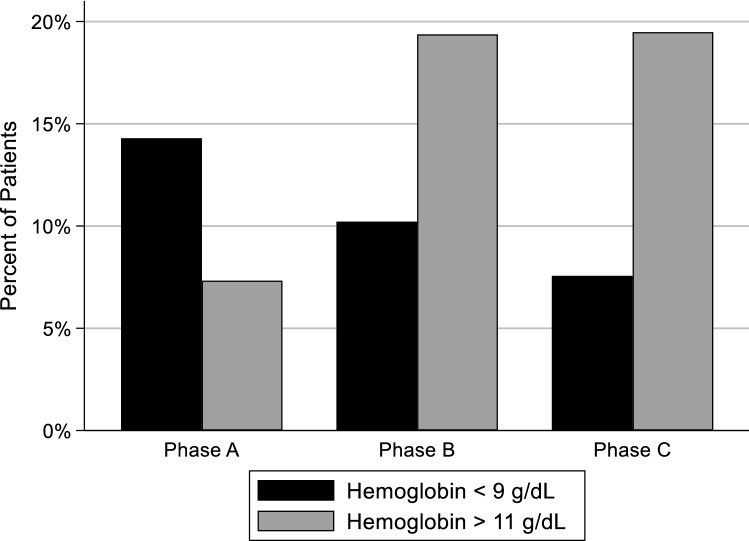
Percentage of patients with Hb < 9 (below target) and Hb > 11 g/dL (above target) in each phase.

The percentage of patient observation below, within, and above range is shown in Fig. 1 and Appendix B (Supplementary material). The percent of patient' observations with Hb < 9 g/dL consistently dropped over the three phases (14.3%, 10.2%, 7.6% in Phases A, B, and C, respectively). The percent of patients with Hb < 9 g/dL in Phase A versus Phase C (14.3% vs 7.6%, respectively) was significantly higher: p = 0.007. The percentage of patients with Hb > 11 g/dL was similar in Phases B and C (19.4% and 19.5%) but significantly higher than in Phase A (7.3%): p < 0.001.

**Figure 2:**
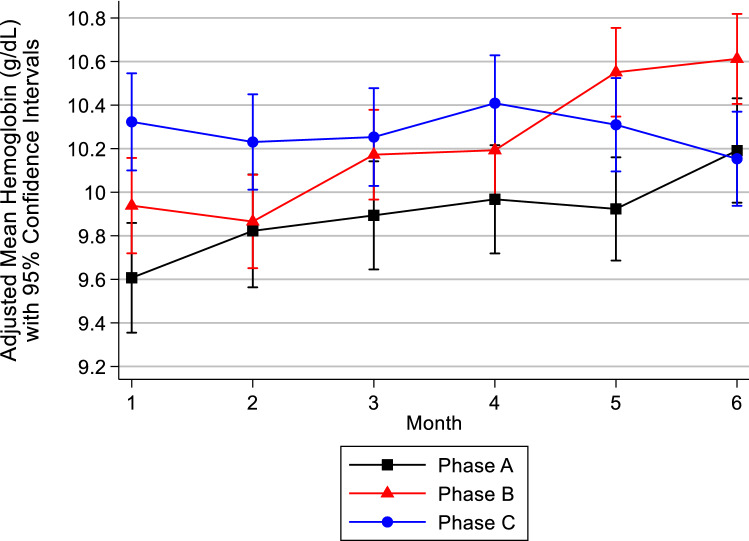
Adjusted Mean Marginal Hb Values by Month Stratified by Phase.

Table 3 presents the adjusted mean marginal effects of Hgb in Phase B and C compared to Phase A. These results are derived from the full multilevel mixed-effects linear regression model (Supplementary material, Appendix C). We found that in Phase C, the adjusted mean marginal effect of Hb was 0.37 g/dL higher; 95% CI (0.23, 0.51), p < 0.001 compared to Phase A. However, phase association with Hb was not constant over the months. Figure 2 depicts the adjusted interaction effects of month and Phase derived from the multi-level mixed-effect regression model. For example, in Phase C compared to Phase A, the Hb is 0.7 g/dL higher on average in Month 1 of the phases (see Fig. 2 and supplementary material, Appendices B and C). Notably, the adjusted mean Hb in Phase C was the most stable over time and higher than the means in Phase A or B.

**Table 3:** Adjusted Mean Marginal Effects from the Mixed-effects regression model with predictors: Phase, month, phase-month interaction, TSAT, Ferritin, TIBC, total administered iron (Venofer + Triferic), and Aranesp.

Variable	Difference in Adjusted (mean) Marginal Effects in Hb (g/dL)	95% CI	p-value with adjusted mean Hb in Phase A as the referent category
Phase A (baseline category)	–	–	
Phase B	0.33	(0.19, 0.46)	0.053
Phase C	0.37	(0.23, 0.51)	< 0.001

We included as predictors the variables: phase, month, the interaction between phase and month, ferritin, TSAT, total iron-binding capacity (TIBC), Aranesp, and Total administered iron (Venofer + Triferic) at each month over time.

## Discussion

The lack of predictive models describing the response of ESA-stimulated erythropoiesis to parental iron administration continues to be a significant hurdle in the pursuit of effective and efficient anemia management in ESRD that balances both iron deficiency, iron overload and variability in demand. For example, ferritin and TSAT levels are inadequate for predicting iron status under iron-release blockage due to inflammation^13^. In addition, ESA pharmacokinetics and temporal dosing patterns generate variability in the dynamic demand for iron for erythropoiesis. The variability is further amplified when ESA dosages become less uniform.

As expected, the introduction of FPC protocol and individualized ESA management resulted in reduction of Hb variability in Phase C. Adjusting mean marginal Hb values by month showed that mean Hb spanned a range of 0.6 g/dL in Phases A and B and exhibited a steady temporal increase. In contrast, in Phase C, mean Hb remained stable and spanned a reduced range of 0.2 g/dL. This analysis shows that after adjusting for key parameters and medications between Phases, the relative stability of mean Hb in Phase C is associated with the novel integration of ferric pyrophosphate citrate and individualized ESA protocols.

The introduction of ferric pyrophosphate citrate in the iron protocol in Phase B led to a decrease in iron sucrose usage. The total amount of monthly parenteral iron was also reduced from 174 mg down to 145 mg (a −16.7% decrease) but the reduction did not meet statistically significance, p = 0.207. Adjusted mean Hb increased significantly from 9.9 g/dL to 10.2 g/dL (p = 0.022) despite similar mean monthly Aranesp doses (97 mcg vs. 95 mcg). Both Ferritin and TSAT were significantly reduced in Phase B (p < 0.05), suggesting a healthy reduction in iron overload. This result supports the findings in^10,14^, which showed the unique mechanism of action and bioavailability for ferric pyrophosphate citrate was more effective in supporting the dynamic demand for iron in the ESA-stimulated erythropoiesis.

The change from standard ESA protocol (Phases A and B) into an individualized ESA management in Phase C, along with the FPC protocol, did not result in significant changes in iron sucrose or the total amount of monthly parenteral iron (iron sucrose plus ferric pyrophosphate citrate). Mean Hb remained similar (10.2 g/dL vs. 10.3 g/dL, p = 0.323) while monthly Aranesp doses saw a 5.2% reduction from the levels in Phases A and B. Ferritin was further reduced by 13.6%, and TSAT rose modestly from 28.5% to 29.8%, p = 0.279. The decrease in ESA demand without a commensurate reduction in Hb supports the findings in^5^, which reported that individualized ESA protocol is more efficient than standard ESA protocols.

The improved Hb outcome in Phase C may also be explained by referring to stable serum EPO profiles in a healthy state. In anemia of ESRD, serum ESA profiles are low and then exhibit a rapid increase and decline following an ESA administration^15^. With longer-acting ESAs, this variability is typically lower. The standard deviation of mean monthly Aranesp in Phase C decreased by 29.4% relative to Phase A. Mean monthly Aranesp standard deviation in Phase C dropped by 29.4% relative to Phase A. A similar reduction between Phases A and C, 30.9%, was observed in the standard deviation of the total amount of monthly parenteral iron. These results raise an interesting question: is it possible that lower variability of ESA and parenteral iron, which is more “physiological,” is associated with improved Hb stabilization and lower drugs?

**Figure 3:**
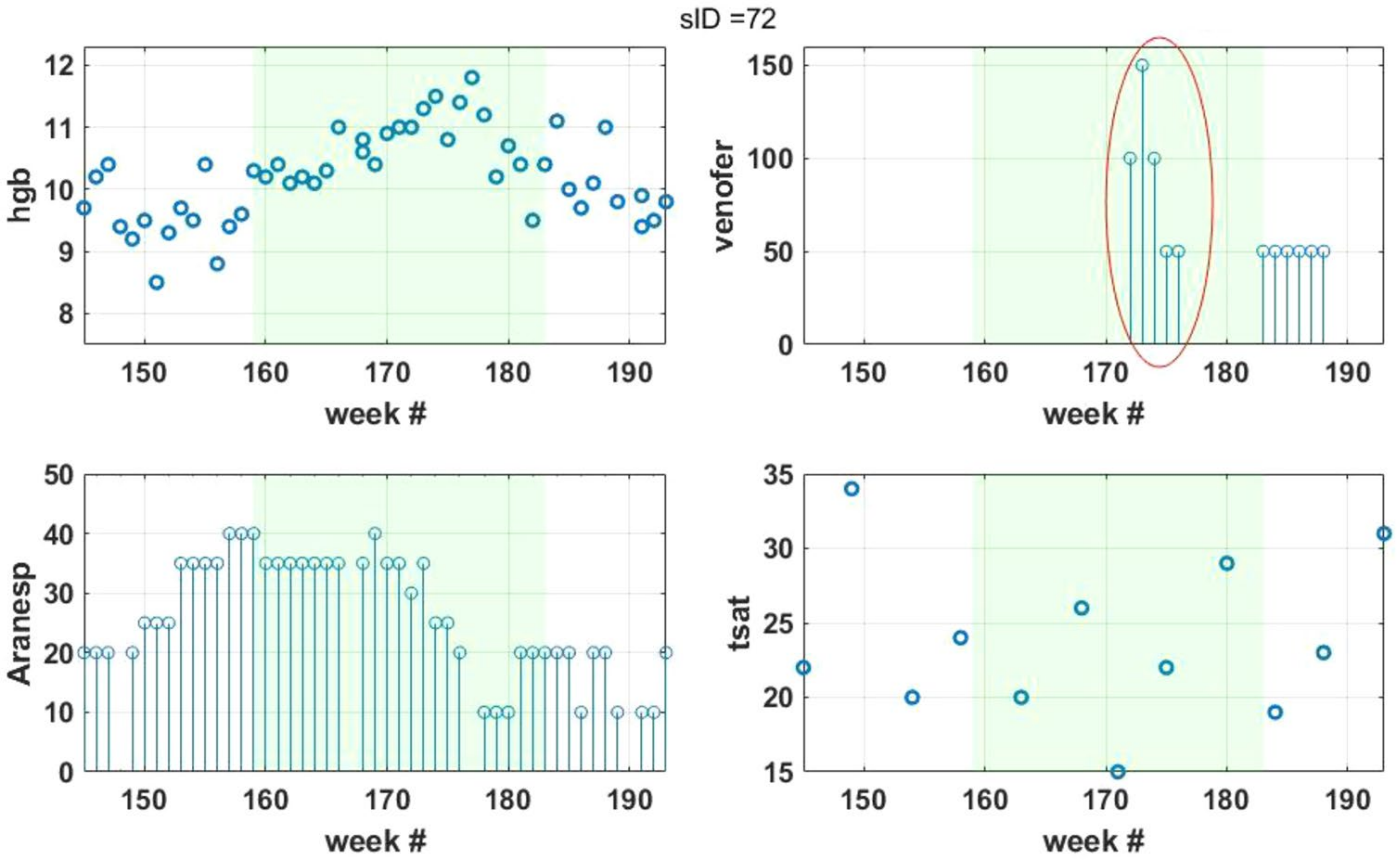
Plot of Phase C (green shaded region) of a single patient showing the temporal crosstalk between the effects of ESA and parenteral iron administration and the resulting Hb response. Displayed values are actual weekly or monthly lab results and ESA doses.

The percentage of patients with Hb below 9 g/dL declined between Phase A and Phases B-C and the percentage of patients with Hb > 11 g/dL increased between Phase A and Phases B-C, p < 0.001. The total parenteral iron dose was similar in all 3 Phases, supporting the notion that ferric pyrophosphate citrate is more effective in supporting erythropoiesis^10,14^. The increase in the percentage of patients within target Hb of 9–11 g/dL between Phases B and C may be attributed to introducing the individualized ESA protocol. The higher percentage of patients with Hb above 11 g/dL in Phase C may also be explained using the temporal change in Hb following the administration of drugs shown in Fig. 3. A relatively stable ESA dose (~ 35 mcg/week) pattern from 4 weeks before the start of Phase C up to week 175 is observed, along with a stable Hb in the 10–10.5 g/dL range. However, a Hb increase reaching near 12 g/dL is observed during weeks 173–178. It is reasonable to assume that this increase is due to the dosing of parenteral IV iron that occurred over weeks 172–176 (Fig. 3, see the circled region in the Venofer panel) during that period. The lack of designed interaction between the ESA and IV iron protocols contrasts with the relationship between erythropoietin and serum iron in erythropoiesis^16^. The constant ESA level was maintained despite the start of Venofer administration and was reduced only after the Hb increase.

Randomized controlled trials (RCT) are the gold standard for testing new interventions and establishing their statistical significance. They are performed in a carefully selected subject population (inclusion/exclusion criteria) in a controlled environment powered to achieve statistical significance. In quality improvement projects, the new intervention is evaluated in a more realistic clinical environment in an unselected patient population in evolving clinical conditions. Compared to RCTs, quality improvement projects do not provide as strong statistical evidence for the superiority of the new intervention but may provide constructive clinical validity. There are several limitations to this continuous quality improvement project. First, not all patients completed all 3 phases of the project. However, the turnover is typical of practice in a real-world setting due to movement between units, transplantation, etc. Second, hemoglobin targets varied across phases from 9–11 g/dL in Phases A-B to 10.2 g/dL in Phase C, complicating the analysis. Third, the FPC protocol recommendations were not always followed in Phases B and C, where a lower TSAT target was implemented, resulting in parenteral IV iron administration. Since iron has a major effect on anemia management, a trial strictly adhering to the iron protocol would strengthen the findings. Finally, the analysis is based on the experiences of a single hemodialysis center. While we found statistically significant differences between phases with our sample size and our cohort was demographically diverse, a future multi-center study on a larger sample size would provide more definitive results on intra-patient variability.

In summary, dialysis units are required to do continuous quality improvement projects and are incentivized to keep Hb levels within a target range. We worked with the dialysis staff to define the problem (high variability with many patients below 9 g/dl). We used our model to measure and analyze the Hb variability. Our protocol was designed to improve (decrease) variability and maintain control of drug (ESA and parenteral iron) doses. The results and processes were discussed with the staff to assess ease of use and barriers.

## Conclusions

This quality improvement project provides evidence that a more patient-specific protocol may overcome some of the limitations of current one-size-fits-all anemia management protocols. Specifically, a protocol of individualized ESA dosing combined with ferric pyrophosphate citrate significantly increased Hb, significantly reduced Ferritin, significantly reduced TSAT (within target) and maintained the total amount of administered iron. In addition, it achieved a reduced Aranesp dose and Aranesp variability despite the increased Hb levels. Our results suggest that ferric pyrophosphate citrate supports more efficient ESA-stimulated erythropoiesis. A protocol of individualized ESA combined with ferric pyrophosphate citrate protocol appears to offer further improvement in efficiency. Future studies are warranted to confirm these findings on a larger, more diverse cohort of ESRD patients.

## Supplementary Information

Supplementary Information.

## Data Availability

The datasets generated during this project are available from the corresponding author on request.

## References

[1] Stauffer ME, Fan T (2014). Prevalence of anemia in chronic kidney disease in the United States. PLoS ONE.

[2] St Peter WL, Guo H, Kabadi S, Gilbertson DT, Peng Y, Pendergraft T, Li S (2018). Prevalence, treatment patterns, and healthcare resource utilization in Medicare and commercially insured non-dialysis-dependent chronic kidney disease patients with and without anemia in the United States. BMC Nephrol..

[3] Babitt JL, Eisenga MF, Haase VH, Kshirsagar AV, Levin A, Locatelli F, Małyszko J, Swinkels DW, Tarng DC, Cheung M, Jadoul M, Winkelmayer WC, Drüeke TB, Conference Participants (2021). Controversies in optimal anemia management: conclusions from a Kidney Disease: Improving Global Outcomes (KDIGO) conference. Kidney Int..

[4] Kotas ME, Medzhitov R (2015). Homeostasis, inflammation, and disease susceptibility. Cell.

[5] Kanbay M, Perazella MA, Kasapoglu B, Koroglu M, Covic A (2010). Erythropoiesis stimulatory agent-resistant anemia in dialysis patients: Review of causes and management. Blood Purif..

[6] Van Wyck DB, Alcorn H, Gupta R (2010). Analytical and biological variation in measures of anemia and iron status in patients treated with maintenance hemodialysis. Am. J. Kidney Dis..

[7] Gaweda AE, Aronoff GR, Jacobs AA, Rai SN, Brier ME (2014). Individualized anemia management reduces hemoglobin variability in hemodialysis patients. J. Am. Soc. Nephrol..

[8] Chait Y, Germain MJ, Hollot CV, Horowitz J (2021). The role of feedback control design in developing anemia management protocols. Ann. Biomed. Eng..

[9] Rostoker G, Vaziri ND (2019). Risk of iron overload with chronic indiscriminate use of intravenous iron products in ESRD and IBD populations. Heliyon.

[10] Gupta A, Lin V, Guss C, Pratt R, Ikizler TA, Besarab A (2015). ferricferric pyrophosphate citrate administered via dialysate reduces erythropoiesis-stimulating agent use and maintains hemoglobin in hemodialysis patients. Kidney Int..

[11] Macdougall IC, White C, Anker SD, Bhandari S, Farrington K, Kalra PA, McMurray JJV, Murray H, Tomson CRV, Wheeler DC, Winearls CG, Ford I (2019). PIVOTAL investigators and committees intravenous iron in patients undergoing maintenance hemodialysis. N. Engl. J. Med..

[12] Collister D, Tangri N (2019). Post-PIVOTAL iron dosing with maintenance hemodialysis. Clin. J. Am. Soc. Nephrol..

[13] Besarab A, Drueke TB (2021). The problem with transferrin saturation as an indicator of iron 'sufficiency' in chronic kidney disease. Nephrol. Dial. Transpl.

[14] Fishbane SN, Singh AK, Cournoyer SH, Jindal KK, Fanti P, Gus CD, Lin VH, Pratt RD, Gupta A (2015). ferricferric pyrophosphate citrate (Triferic™) administration via the dialysate maintains hemoglobin and iron balance in chronic hemodialysis patients. Nephrol. Dial. Transpl..

[15] Fishbane S, Besarab A (2007). Mechanism of increased mortality risk with erythropoietin treatment to higher hemoglobin targets. Clin. J. Am. Soc. Nephrol..

[16] Li H, Ginzburg YZ (2010). Crosstalk between iron metabolism and erythropoiesis. Adv. Hematol..

