# Supplementary material for: Individualized anemia management enhanced by ferric pyrophosphate citrate protocol
Source: Sci Rep. 2022 Nov 22;12:20122. doi: 10.1038/s41598-022-23262-1 (PMC9684411; doi:10.1038/s41598-022-23262-1)
Supplement: Supplementary file 1 — Supplementary Information. [file 41598_2022_23262_MOESM1_ESM.docx]

**Appendix A: Anemia Management Protocols**

Phase A: ESA and IV iron titration algorithms are proprietary "if-then" decision rules. Hb target was 9-11 g/dL, and TSAT target was >30%. If Hb > 11 g/dl, ESA was held.

Phase B: ESA titration algorithm was same as in Phase A. Under the ferric pyrophosphate citrate protocol, IV iron was not administered unless TSAT was below its target of 25%.

Phase C: Under the individualized ESA management, Hb target was 10.2 g/dL. The foundation of the ESA titration algorithm is described in [8] and references therein. ESA was not held at Hb > 11 g/dL.

**Appendix B: Hemoglobin Values by Phase**

| Hemoglobin value | Phase A  N = 273  observations from 54 patients | Phase B  N = 382  observations from 78 patients | Phase C  N = 344  observations from 66 patients |
| --- | --- | --- | --- |
| < 9 g/dL | 39 (14.3%) | 39 (10.2%) | 26 (7.6%) |
| Between 9 and 11 g/dL | 214 (78.4%) | 269 (70.4%) | 251 (73.0%) |
| > 11 g/dL | 20 (7.3%) | 74 (19.4%) | 67 (19.5%) |

In Appendix B, the number of hemoglobin observations below 9 g/dL and above 11 g/dL are given and the differences between phases were statistically significant (p<0.001).

# **Appendix C: Results from the Multi-Level Mixed-effects regression model for the outcome of Hemoglobin with predictors: Phase, month, phase-month interaction, TSAT, Ferritin, TIBC, total administered iron (Venofer + Triferic), and Aranesp**

| **Covariate** | **Coefficient** | **95% Confidence**  **Interval** | **P-value** |
| --- | --- | --- | --- |
| Phase | 0 |  |  |
| Phase A* | 0.332 | (-0.004 to 0.667) | 0.053 |
| Phase B | 0.716 | (0.378 to 1.055) | <0.001 |
| Phase C |  |  |  |
| Month |  |  |  |
| 1* | 0 |  |  |
| 2 | 0.216 | (-0.141 to 0.572) | 0.236 |
| 3 | 0.287 | (-0.066 to 0.639) | 0.111 |
| 4 | 0.360 | (0.008 to 0.713) | 0.045 |
| 5 | 0.316 | (-0.029 to 0.661) | 0.072 |
| 6 | 0.584 | 0.238 to 0.930) | 0.001 |
| Phase & Month interaction |  |  |  |
| Phase B x Month 2 | -0.289 | (-0.758 to 0.180) | 0.228 |
| Phase B x Month 3 | -0.053 | (-0.515 to 0.409) | 0.823 |
| Phase B x Month 4 | -0.106 | (-0.572 to 0.360) | 0.655 |
| Phase B x Month 5 | 0.296 | (-0.162 to 0.754) | 0.205 |
| Phase B x Month 6 | 0.089 | (-0.371 to 0.549) | 0.704 |
| Phase C x Month 2 | -0.308 | (-0.783 to 0.167) | 0.204 |
| Phase C x Month 3 | -0.357 | (-0.830 to 0.117) | 0.140 |
| Phase C x Month 4 | -0.275 | (-0.746 to 0.196) | 0.253 |
| Phase C x Month 5 | -0.330 | (-0.793 to 0.133) | 0.163 |
| Phase C x Month 6 | -0.754 | (-1.219 to -0.289) | 0.001 |
| TSAT per unit | 0.000 | (-0.004 to 0.005) | 0.854 |
| Ferritin per unit | -0.000 | (-0.000 to -0.000) | 0.001 |
| TIBC per unit | 0.003 | (0.002 to 0.005) | <0.001 |
| Total Iron per unit | 0.000 | (-0.000 to 0.001) | 0.571 |
| Aranesp per unit | -0.004 | (-0.005 to -0.003) | <0.001 |
| Constant | 9.384 | (8.860 to 9.908) | <0.001 |

* Indicates the baseline (referent) category
